# Mitochondrial Permeability Transition and Cell Death: The Role of Cyclophilin D

**DOI:** 10.3389/fphys.2013.00076

**Published:** 2013-04-11

**Authors:** Sabzali Javadov, Andrey Kuznetsov

**Affiliations:** ^1^Department of Physiology, School of Medicine, University of Puerto RicoSan Juan, PR, USA; ^2^Cardiac Surgery Research Laboratory, Department of Cardiac Surgery, Innsbruck Medical UniversityInnsbruck, Austria

**Keywords:** mitochondria, permeability transition pore, cyclophilin D, cell death

## Abstract

Mitochondria serve as a “powerhouse” which provides near 90% of ATP necessary for cell life. However, recent studies provide strong evidence that mitochondria also play a central role in cell death. Mitochondrial permeability transition (mPT) at high conductance in response to oxidative or other cellular stresses is accompanied by pathological and non-specific mPT pore (mPTP) opening in the inner membrane of mitochondria. Mitochondrial PTP can serve as a target to prevent cell death under pathological conditions such as cardiac and brain ischemia/reperfusion injury and diabetes. On the other hand, mPTP can be used as an executioner to specifically induce cell death thus blocking tumorigenesis in cancer diseases. Despite many studies, the molecular identity of the mPTP remains unclear. Cyclophilin D (CyP-D) plays an essential regulatory role in pore opening. This review will discuss direct and indirect mechanisms underlying CyP-D interaction with a target protein of the mPTP complex. Understanding of the mechanisms of mPTP opening will be helpful to further develop new pharmacological agents targeting mitochondria-mediated cell death.

## Mitochondrial Permeability Transition and Cell Death

Studies over the past 30 years demonstrated that, in addition to their role in cell life, mitochondria are the main executioners of cell death in response to oxidative stress. Accumulation of ROS along with Ca^2+^ overload induces is mitochondrial permeability transition (mPT) that is associated with non-selective pathological PT pore (mPTP) opening in the inner membrane of mitochondria (IMM). Opening of the mPTP is accompanied by loss of the mitochondrial membrane potential and proton gradient across the IMM. At low electrochemical potential, *F*_0F1_-ATPase induces ATP hydrolysis in an attempt to maintain the mitochondrial membrane potential, and adenine nucleotide translocase (ANT) functions in a “reverse mode”, transporting ATP to the matrix. Mitochondrial PT can occur at low and high conductance leading to reversible of irreversible consequences. Reversible mPTPs are permeable to ions and solutes with the molecular mass <300 Da, and do not induce notable matrix swelling (Brenner and Moulin, 2012). This mode may be important in regulation of mitochondrial Ca^2+^ homeostasis since mitochondrial Ca^2+^ efflux is inhibited by the immunosupressor cyclosporine A (CsA) in various cells including cardiomyocytes (Altschuld et al., 1992). Furthermore, the low-conductance mode can initiate mitochondrial depolarization spikes generating and conveying calcium signals (waves) from one mitochondrion to another (Ichas et al., 1997). In a high conductance mode, solutes, water, and ions with the molecular mass up to ∼1.5 kD enter through the mPTP thus enhancing colloid-osmotic pressure in the matrix. This causes rupture of the outer membrane of the mitochondria (OMM) leading to cell death via apoptosis and/or necrosis depending on the ATP level in cells. Opening of the mPTP is regulated by ions (*P*_i_, H^+^, Ca^2+^, Mg^2+^), ROS, adenine nucleotides, ubiquinones (Halestrap et al., 1998; Bernardi, 1999; Crompton, 1999), and many other factors.

## Cyclophilin D is the Only Defined mPTP Component

Although mPT induction has been broadly accepted as a well-known phenomenon the molecular identity of the mPTP still remains elusive. Initially three proteins, ANT in the IMM, voltage-dependent anion channel (VDAC or porin) in the OMM, and cyclophilin D (CyP-D) in the matrix were proposed as the main structural components of the mPTP. In addition, the benzodiazepine receptor, hexokinase, creatine kinase, Bcl2, phosphate carrier (PiC), and other proteins may play regulatory roles in pore opening (Weiss et al., 2003). Later, genetic studies conducted in knock-out mice demonstrated that mitochondria containing neither VDAC nor ANT were still susceptible to Ca^2+^-induced mPTP induction therefore excluding the role of these proteins as the essential structural components of the mPTP (Kokoszka et al., 2004; Basso et al., 2005; Baines et al., 2007). However, mitochondria isolated from Cyp-D^-/-^ mice were more resistant to mPTP opening than wild-type mice and exhibited mPT induction at higher [Ca^2+^], and less cell death in response to oxidative stress (Baines et al., 2005; Nakagawa et al., 2005). In addition, mPTP-mediated cell death preferably occurred through necrosis rather than apoptosis as CyP-D^-/-^ cells were resistant to necrotic stimuli but demonstrated similar sensitivity to apoptotic factors as wild-type cells (Nakagawa et al., 2005). It must be noted that although genetic studies revealed VDAC and ANT as the non-essential pore components, many questions related to the role of these proteins in mPT induction remain unresolved. Recently, PiC was identified as an essential component of the mPTP although studies on PiC knock-out mice are still required to validate these data (Varanyuwatana and Halestrap, 2012). The presence of a large number of proteins in the IMM and the dynamic structure of the pore complex apparently make difficult to uncover its molecular identity. Here we will focus on CyP-D, which on the basis of multiple genetic and biochemical studies has been accepted as a key regulator and component of the pore opening. CyP-D belongs to cyclophilins known as peptidyl-prolyl cis–trans isomerases, a family of proteins that catalyze the *cis-trans* isomerization of peptydyl-prolyl bonds, and possess chaperone activity to regulate protein folding. There are seven major cyclophilin isoforms found in subcellular compartments including the cytoplasm (CyP-D, CyP-NK, CyP-40), endo(sarco)plasmic reticulum (CyP-B, CyP-C), nucleus (CyP-E), and mitochondria (CyP-D) (Lee and Kim, 2010). Notably, individual cyclophilins can have distinct effects on cell survival under pathological conditions. Studies performed on various cancer models and tissue samples from patients demonstrated that overexpression of CyP-A stimulates cancer cell growth (reviewed in Lee and Kim, 2010). On the other hand, expression of CyP-D, a soluble matrix protein, is associated with mPTP opening and cell death during ischemia/reperfusion in the heart and brain. CyP-D is a nuclear encoded protein widely expressed in all mammalian tissues. It contains a mitochondrial targeting presequence which is cleaved after its translocation into the matrix (Connern and Halestrap, 1992). Homozygous CyP-D knock-out mice exhibit normal phenotype (Basso et al., 2005; Nakagawa et al., 2005) although develop insulin resistance (Rieusset et al., 2012). In addition to its role in pore opening, CyP-D has been shown to catalyze folding of newly imported proteins in the matrix of mitochondria (Matouschek et al., 1995). Recent studies on human SH-SY5Y neuroblastoma cells demonstrated that CyP-D can also act as a redox sensor in mitochondria of mammalian cells (Linard et al., 2009), and regulate Ca^2+^ exchange between endoplasmic reticulum and mitochondria (Rieusset et al., 2012).

## The Role of CyP-D in Pore Opening

**Figure 1:**
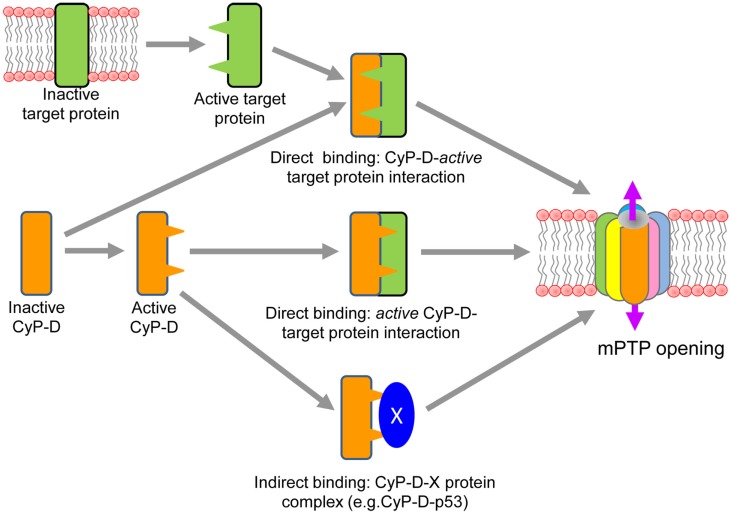
**Proposed direct and indirect mechanisms of CyP-D interaction with a target protein of the mPTP complex**.

The mechanisms of interaction of CyP-D with a target protein(s) in the IMM and the induction of conformational changes of the target protein to form the mPTP complex remain unrevealed. Importantly, the translocation of CyP-D from the matrix to the IMM and its interaction with a target protein to induce pore opening in response to oxidative stress can occur through both *direct* and *indirect* mechanisms (Figure 1). *Direct binding* of CyP-D to a target protein in the IMM can be triggered by activation of the latter in response to oxidative stress. Oxidative stress can induce conformational changes of the target protein by chemical modification and/or alterations in the inner membrane topography due to increased matrix swelling. Most, if not all, previous studies were focused on ANT as a target protein interacting with CyP-D to initiate the pore opening. Initial studies provided strong evidence that Ca^2+^-triggered conformational change of the ANT is a key step in mPTP opening which is facilitated by CyP-D binding. GST-CyP-D pull-down and co-immunoprecipitation studies on isolated mitochondria revealed CsA-sensitive binding of CyP-D to ANT (Crompton et al., 1998; Woodfield et al., 1998). Also, oxidative stress sensitizes the mPTP to [Ca^2+^] by antagonizing adenine nucleotide binding, and enhances CyP-D binding to the ANT (McStay et al., 2002). Chemical modifications of three cysteine residues (Cys56, Cys159, and Cys256) in ANT in response both to oxidative stress and thiol reagents were shown to be associated with a conformational change of the exchanger (Majima et al., 1993). Two distinct thiol groups have been identified to participate in the modulation of mPTP activity (Costantini et al., 1996), and cysteine residues in the ANT may represent these thiol groups that regulate the binding affinity of the ANT for CyP-D and ADP (Halestrap et al., 1997). Consequently, oxidative stress or thiol reagents have been shown to induce cross-linking of two matrix facing cysteine residues (Cys56 and Cys159) of ANT that modulate mPTP activity through the CyP-D-ANT interaction (Halestrap and Brenner, 2003). Direct binding of CyP-D to a target protein in the IMM can also occur through activation of the former. In fact, Cys203 residue of Cyp-D has been shown to play a crucial role in oxidative stress induced activation of mPTP in mouse embryonic fibroblasts (Nguyen et al., 2011). CyP-D can be activated in the matrix due to post-translational modification, which may facilitate its translocation to the IMM and initiate mPT (Figure 1). Moreover, CyP-D can undergo post-transitional modifications (phosphorylation, nitrosylation, acetylation, etc.) on specific site(s) which would increase its activity to interact with a target protein. However, at present, there are rather few studies directly showing post-translational modifications of CyP-D. Recent studies also discovered that acetylation of CyP-D due to inhibition of the mitochondrial isoform of sirtuins, SIRT 3, a NAD^+^ dependent deacetylase, increased interaction of CyP-D with ANT (Shulga and Pastorino, 2010). Furthermore, CyP-D acetylation was associated with reduced SIRT3 expression and increased pore opening in heart failure induced by transverse aortic constriction (Hafner et al., 2010) and myocardial infarction (Parodi-Rullan et al., 2012) in rodents. In addition, significant fraction of GSK-3beta has been shown to be co-localized with CyP-D in mitochondria, suggesting thus a potential regulatory role for GSK-3beta in pore opening. Active GSK is shown to phosphorylate CyP-D in an ERK1/2-dependent manner, and phospho-CyP-D^Ser/Thr^ promoted depolarization of mitochondria and pore opening (Rasola et al., 2010). Conversely, pharmacological inhibition of GSK-3beta prevented the phosphorylation of CyP-D, which may lead to the inhibition of the mPT in murine tubular epithelial cells (Bao et al., 2012). Post-translational modification of CyP-D induced by nitrosylation may also be important in regulating of the mPTP. *In vitro* studies using proteins and cells revealed that both NO and ONOO^−^ can affect ANT and increase mPT in a CsA-sensitive manner (Vieira et al., 2001), suggesting a key role of nitrosylation in the activation of pore opening. Nitric oxide can induce or inhibit the mPT depending on its concentration in the cell (Burwell and Brookes, 2008). Recent studies demonstrated that treatment of heart homogenates with GSNO resulted in S-nitrosylation of CyP-D on cysteine-203 (Kohr et al., 2011). Increased nitration of CyP-D as well as VDAC and ANT on tyrosine was found in mitochondria of neurons after cortical injury which was associated with elevated ROS production and cell death (Martin et al., 2011). However, it is not clear yet how nitrosylated CyP-D interacts with the target protein to induce mPT. Recent *in vitro* studies demonstrated that CyP-D association to the lateral stalk of *F*_0F__1_-ATP synthase modulates the activity of the complex, and the ATP synthase-CyP-D interactions were modulated by *P*_i_ and CsA, respectively, increasing and decreasing CyP-D binding to the enzyme (Giorgio et al., 2009). Interestingly, *P*_i_ was specifically required for PTP desensitization by CsA or by CyP-D ablation (Basso et al., 2008) as well as for inhibition of mPTP by blocking the complex I (Li et al., 2012).

*Indirect binding* of CyP-D to a target protein(s) in the IMM can occur through its interaction with other proteins in the matrix. Most recent studies demonstrated that in response to oxidative stress induced by brain ischemia/reperfusion injury p53, a tumor suppressor protein, accumulates in the mitochondrial matrix and triggers mPTP opening and necrosis by physical interaction with CyP-D (Vaseva et al., 2012). Conversely, reduction of p53 levels or treatment of mice with CsA prevented the p53-Cyp-D complex opening which was associated with effective stroke protection (Vaseva et al., 2012). Likely, p53 triggers translocation of CyP-D to the IMM and therefore facilitates the pore opening through interaction with a pore protein(s). However, the study demonstrated no regulation of calcium-dependent MPTP opening by p53. It is not clear how p53-CyP-D interaction senses and induces mPTP opening in a Ca^2+^-independent manner (Karch and Molkentin, 2012). Notably, binding of CyP-D to a matrix protein in cancer cells may have an opposite effect, leading to inhibition of the mPTP. Also, it has been demonstrated that abundant expression of Hsp60 in mitochondria of tumor cells is associated with increased levels of the Hsp60-CyP-D complexes and reduced mPTP opening (Ghosh et al., 2010). Conversely, Hsp90 antagonists directed to mitochondria caused severe mitochondrial dysfunction and selective tumor cell death inhibiting the interaction of Hsp90 with CyP-D (Kang et al., 2007). Likewise, interaction of CyP-D with Bcl2 has been shown to exert an anti-apoptotic effect, and CsA, disrupted the CyP-D-Bcl2 interaction. The anti-apoptotic effect of CyP-D in some cancer cells which overexpress the protein can be explained by CyP-D-Bcl2 interaction to suppress apoptosis in these cells (Eliseev et al., 2009).

Thus, accumulating data suggest that activation of CyP-D and its interaction with the mPTP complex can occur through different mechanisms including (i) post-translational modification of the protein, (ii) direct interaction with an active target protein, and/or (iii) indirectly via binding to a matrix protein.

## Conclusion

Irreversible mPTP opening acts as a target and executioner of cell death under pathological conditions such as cardiac and brain ischemia/reperfusion, diabetes, and cancer. Although mPT is a well-known phenomenon, the molecular identity of the mPTP complex is still unidentified. Existing studies provide strong evidence that CyP-D plays a regulatory role in mPT, and understanding the mechanism(s) of CyP-D activation and its interaction with the mPTP complex is important in developing new pharmacological agents to modulate mitochondria-mediated cell death.

## Conflict of Interest Statement

The authors declare that the research was conducted in the absence of any commercial or financial relationships that could be construed as a potential conflict of interest.
